# Genetic Analysis of Agronomic Traits and Grain Iron and Zinc Concentrations in a Doubled Haploid Population of Rice (*Oryza sativa* L.)

**DOI:** 10.1038/s41598-020-59184-z

**Published:** 2020-02-10

**Authors:** Mark Ian C. Calayugan, Andrea Kariza Formantes, Amery Amparado, Gwen Iris Descalsota-Empleo, Chau Thanh Nha, Mary Ann Inabangan-Asilo, Zin Mar Swe, Jose E. Hernandez, Teresita H. Borromeo, Antonio G. Lalusin, Merlyn S. Mendioro, Ma. Genaleen Q. Diaz, Celia B. dela Viña, Russell Reinke, B. P. Mallikarjuna Swamy

**Affiliations:** 1 International Rice Research Institute (IRRI), DAPO Box 7777 Metro Manila, Philippines; 2 University of the Philippines Los Baños, Laguna, 4031 Philippines; 3 University of the Southern Mindanao, Kabacan, Cotabato 9407 Philippines; 4 Cuu Long Delta Rice Research Institute (CLRRI), Cần Thơ, Vietnam; 5 Department of Agriculture, Yezin, Myanmar

**Keywords:** Biotechnology, Plant sciences

## Abstract

The development of micronutrient dense rice varieties with good agronomic traits is one of the sustainable and cost-effective approaches for reducing malnutrition. Identification of QTLs for high grain Fe and Zn, yield and yield components helps in precise and faster development of high Fe and Zn rice. We carried out a three-season evaluation using IR05F102 x IR69428 derived doubled-haploid population at IRRI. Inclusive composite interval mapping was carried out using SNP markers and Best Linear Unbiased Estimates of the phenotypic traits. A total of 23 QTLs were identified for eight agronomic traits and grain Fe and Zn concentration that explained 7.2 to 22.0% PV. A QTL by environment interaction analysis confirmed the stability of nine QTLs, including two QTLs for Zn on chromosomes 5 and 12. One epistatic interaction for plant height was significant with 28.4% PVE. Moreover, five QTLs were identified for Fe and Zn that harbor several candidate genes, e.g. *OsZIP6* on QTL *qZn*_*5.1*_. A number of QTLs were associated with a combination of greater yield and increased grain Zn levels. These results are useful for development of new rice varieties with good agronomic traits and high grain Zn using MAS, and identification of genetic resources with the novel QTLs for grain Zn.

## Introduction

Rice constitutes a significant portion of the daily diet particularly in developing countries of Asia, supplying 50–80% of the daily caloric intake, proteins, minerals and vitamins etc^1,2^. The annual global rice production of 700 million tons was produced on an area of 158 million hectares in 2017^2^. However, rice production must be increased by additional 8–10 million tons each year and there is a strong impetus to improve the nutritional value with multiple essential vitamins and minerals to meet food and nutritional demands of a growing population^2^. Thus, there is an urgent need to breed for rice varieties with improved yield and nutritional value for sustaining global food and nutritional security^3,4^_._

Micronutrients such as Fe and Zn are highly essential for the normal growth and development of both plants and animals^5^. The recommended human intake of Fe and Zn is 15 mg per day^6^. However, it is rarely met by rice-based diets consumed in Asian populations^7^. Fe and Zn deficiencies significantly impact human health causing anemia, stunting, decreased immunity, poor cognitive development^8^. These have been recognized as global health concerns and need to be addressed on a priority basis^9^. Dietary diversification, food fortification, supplementation and biofortification have been advocated and are being employed to address malnutrition. Among these, biofortification of staple crops is in the forefront because of its affordability and easy access to the needy and targeted vulnerable populations, and the scale and impact that can be achieved by this intervention will be huge^10^.

Modern high-yielding rice varieties are poor source of nutrients in their polished form^11^. However, the huge genetic variability in grain mineral nutrients available in the rice germplasm can be exploited to develop biofortified high yielding rice varieties^12^. Even though genetic complexity and G x E were reported to be a hindrance for improving mineral content with high yield, much progress has been achieved in elucidating the genetic basis and G x E effect on grain micronutrient accumulation, yield and yield related traits in rice^13,14^.

Identification of new donors for agronomic, yield and mineral nutrient traits, dissecting their genetic basis and using them to diversify the breeding population is a continuous process in developing high-yielding and nutritious rice varieties^15–17^. Several QTLs and genes have been identified and successfully used in rice improvement. Marker-Assisted Breeding (MAB) and Genomic Selection (GS) are powerful approaches to improve multiple complex traits^18,19^. The mainstreaming of mineral nutrients (i.e. including nutritive value as a standard selection criterion within breeding programs), especially grain Zn in staple crops has been well recognized and the application of genomics can enhance the rate of genetic gain^10,20^.

Doubled haploid lines (DH) are important genetic resources for mapping QTLs because of their rapid development with less deleterious alleles, minimal background noise and these fixed lines can be readily evaluated across years and locations^21^. DH populations together with Single Nucleotide Polymorphisms (SNPs) are an ideal combination for QTL mapping. They are cost-effective to assay using automated platforms, while allele calling, data analysis, and the database is straightforward due to their bi-allelic nature^22^. In previous studies several DH populations were genetically characterized for yield, yield components and grain mineral elements^23,24^. These DH populations utilized *japonica* type rice cultivars as donors in which grain Zn ranged from 10.37 to 18.00 ppm in brown rice form^18,24,25^. On the other hand, other studies used Backcross Introgression Lines (BILs) derived from *O. sativa ‘Nipponbare’* and *O. meridionalis* W1627^26^, *O. sativa* cv. Swarna x *O. nivara*^27^, and *Oryza sativa* x *O. rufipogon*^28^.

Genomics based breeding approaches including MAS are becoming more common and to be efficient, research breeding programs need robust and stable QTLs across environments and genetic backgrounds^29^. Genotype by environment interactions (GEI) are the expression of QTLs and GEI QTLs are important as they significantly influence the total phenotypic variance and additive effect of the main effect QTL^30^. Moreover, haplotype-based allele mining was used to detect allelic variation in genes controlling agronomic traits^31^. On the other hand, gene pyramiding is important in improving the efficiency of biofortification breeding with SNP chip technology, allowing the development of lines with simultaneously introgressed small-effect QTLs for the precise development of genetic stocks^32^.

We characterized a DH population for yield, yield related traits and grain mineral elements, identified QTLs and candidate genes for yield and yield components, grain Fe and Zn; analyzed epistatic interactions, QTL x environment interactions, QTL pyramiding effects and haplotype analyses of major QTLs for YLD and Zn.

## Results

### Phenotypic analysis in DH population

**Figure 1:**
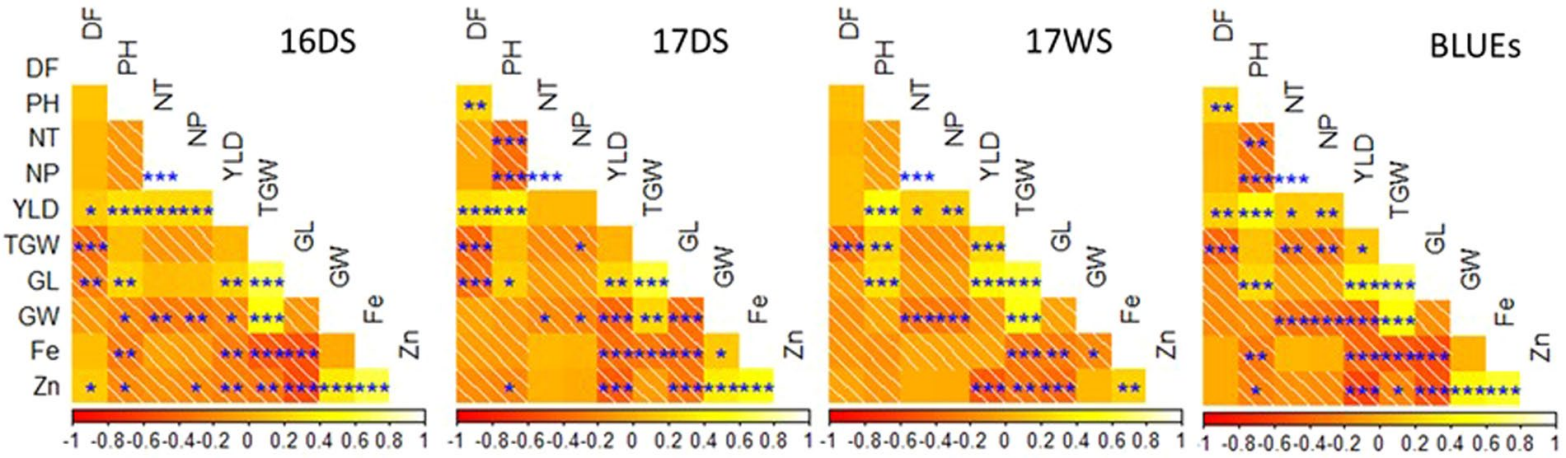
Correlations among agronomic traits and grain Fe and Zn concentrations in DH population. *Indicates significance at *p* ≤ 0.05. **Indicates significance at *p* ≤ 0.01. ***Indicates significance at *p* ≤ 0.001. †DF: days to flowering (days); PH: plant height (cm); NT: number of tillers; NP: number of panicles; YLD: yield (kgha^−1^); TGW: thousand grain weight (g); GL: grain length (mm); GW: grain width (mm); Fe: Iron (ppm); and Zn: Zinc (ppm).

**Figure 2:**
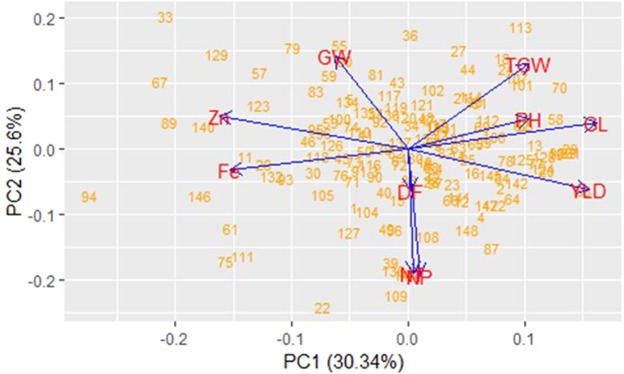
Principal component analysis in DH population using the two PC with highest proportion of variance. Numbers inside the box correspond to DH lines.

The DH population showed wide variability for all traits measured in all three seasons (Supplementary Fig. 1). For agronomic traits, DF ranged from 71.7 to 106.2 days with a mean of 86.9 days. PH ranged from 70.1 to 127.7 cm with a mean of 95.5 cm while YLD ranged from 2669.9 to 7409.1 kg ha^−1^ with a mean of 5446.1 kg ha^−1^. GL ranged from 6.4 to 10.3 mm with a mean of 9.02 mm and GW ranged from 1.8 to 2.3 mm with a mean of 1.97 mm. In grain Fe and Zn concentrations, Fe ranged from 2.8 to 5.8 ppm with mean of 4.1 ppm while Zn ranged from 8.7 to 19.7 ppm with a mean of 12.6 ppm (Table 1). The parent IR05F102 exhibited higher values for agronomic traits NT, NP, and YLD in all three seasons while IR69428 exhibited higher values for DF, PH, TGW, GW, Fe and Zn across three seasons and BLUEs. Coefficient of variation for agronomic traits ranged from 2.88% to 19.20%. Low CVs (<10%) were recorded for DF, PH, TGW, GL, and GW. Genotypic effects were highly significant and heritability values were high (0.52–0.98) for all agronomic traits and grain Zn concentration while lower heritabilities were observed for Fe, *viz*. 0.22 in S3 and 0.49 in S1 (Table 1). Pearson’s correlation co-efficient between BLUEs and the three season’s data showed significant positive correlations ranging between 0.52 and 0.97. Of the 45 possible correlations; 27 were significant in S1, with 12 positively correlated and 15 negatively correlated; while 26 were significant in S2, with 11 positively correlated and 15 negatively correlated; and 20 were significant in S3, of which 11 were positively correlated and nine were negatively correlated. A total of 29 correlations were significant using BLUEs, 13 of them were positive and 16 were negative (Fig. 1, Supplementary Table 1). Consistent strong positive correlations (p < 0.001) were observed between NT & NP, PH & YLD, PH & GL, YLD & GL, TGW & GW, Zn & GW, and Zn & Fe. Meanwhile, strong negative correlations were observed between DF & TGW, GW & NT, GW & NP, GW & YLD. The first two principal components (PC) accounted for 55.94% of the total variation (Fig. 2). PC1 contributed 30.34% of the total variation while PC2 contributed 25.60%. For PC1, GL and YLD showed the highest positive loadings (0.46 and 0.44) among the agronomic traits analyzed, and Fe and Zn exhibited negative loadings (−0.43 and −0.46). This indicates that GL, YLD, Fe and Zn contributed most of the variation in PC1 (Supplementary Table 2). In PC2, TGW and GW contributed most of the variation with loadings of 0.37 and 0.40, respectively.

**Table 1:** Phenotypic variation for agronomic traits, Fe and Zn concentration in DH population.

Trait	Season	Parents		DH lines		ANOVA		
		IR05F102	IR 69428	Range	Mean ± SE	CV (%)	Genotypic effects	H^2^
DF (days)	S1	87	96.5	75.0–107.5	83.74 ± 0.53	7.63	13.4*	0.93
	S2	89	100	75.0–109.0	87.18 ± 0.50	6.99	16.4**	0.94
	S3	91	94.5	72.0–108.0	90.18 ± 0.45	6.1	46.9**	0.98
	BLUEs	88.5	94.5	71.7–106.2	86.98 ± 0.45	6.25	—	—
PH (cm)	S1	88.2	93.4	71.2–130.1	93.98 ± 0.74	9.6	6.8**	0.85
	S2	96.1	100.8	65.5–122.0	92.69 ± 0.77	10.08	6.4**	0.85
	S3	102.3	101.5	67.2–129.8	99.50 ± 0.91	10.98	4.5**	0.78
	BLUEs	95.4	98.9	70.1–127.7	95.48 ± 0.72	9.11	—	—
NT	S1	14.5	10	9.3–22.89	15.42 ± 0.18	14.02	2.1**	0.52
	S2	11.8	7.8	9.5–18.5	13.29 ± 0.15	13.99	2.8**	0.63
	S3	11.6	7.3	7.7–17.0	12.13 ± 0.16	16.33	2.2**	0.55
	BLUEs	12.7	8.4	10.1–18.7	13.87 ± 0.13	11.22	—	—
NP\	S1	13.1	9	9.0–20.89	14.18 ± 0.17	14.57	2.2**	0.53
	S2	11.6	7.8	9.3–18.5	13.08 ± 0.15	13.89	2.7**	0.61
	S3	11.6	7.1	7.5–16.83	11.95 ± 0.17	17.03	2.3**	0.57
	BLUEs	12.2	8	9.5–17.9	13.32 ± 0.13	11.5	—	—
YLD (kg ha^−1^)	S1	8340.6	2186.5	3011.6–13163.6	6856.35 ± 159.02	28.22	3.0**	0.66
	S2	8018.6	4797.2	3022.4–9767.3	6474.54 ± 121.15	22.76	5.8**	0.83
	S3	3605.3	765.2	450.1–5779.3	3095.04 ± 85.37	33.44	4.5**	0.78
	BLUEs	6425.1	3154.4	2669.9–7409.1	5446.10 ± 85.93	19.2	—	—
TGW (g)	S1	21	30.1	16.1–31.3	25.25 ± 0.21	10.25	2.7**	0.62
	S2	27.7	29	16.0–29.7	23.61 ± 0.19	9.95	12.0**	0.91
	S3	26.4	26.8	15.6–28.2	23.23 ± 0.20	10.44	9.2**	0.89
	BLUEs	27.4	28.7	16.6–29.4	24.05 ± 0.19	9.43	—	—
GL (mm)	S1	8.1	8.1	6.4–10.6	9.04 ± 0.06	8.31	18.7**	0.94
	S2	9.6	8.2	6.2–10.3	8.94 ± 0.06	8.46	14.9**	0.93
	S3	9.6	8.2	6.6–10.4	9.10 ± 0.06	8.16	13.4**	0.92
	BLUEs	9.1	8.2	6.4–10.3	9.02 ± 0.06	8.05	—	—
GW (mm)	S1	1.8	2.3	1.8–2.3	1.94 ± 0.006	3.79	9.3**	0.87
	S2	2.1	2.4	1.96–2.3	2.05 ± 0.005	2.7	4.4**	0.7
	S3	2	2.4	1.7–2.4	1.92 ± 0.006	4.06	4.1**	0.7
	BLUEs	1.9	2.4	1.8–2.3	1.97 ± 0.006	2.88	—	—
Fe (ppm)	S1	4	3.9	2.3–5.6	3.71 ± 0.05	15.72	1.9**	0.49
	S2	3.6	5.6	3.2–7.5	5.11 ± 0.06	14.55	1.6**	0.35
	S3	2.7	1.7	1.4–6.6	3.36 ± 0.06	23.05	1.3*	0.22
	BLUEs	3.6	3.9	2.8–5.8	4.06 ± 0.04	12.55		
Zn (ppm)	S1	14.4	23.5	7.8–26.9	13.47 ± 0.27	24.16	8.0**	0.87
	S2	12.4	13.7	9.5–20.6	13.85 ± 0.17	14.68	3.5**	0.71
	S3	9.4	9.2	6.8–21.6	10.63 ± 0.18	20.45	3.9**	0.75
	BLUEs	11.9	14.8	8.7–19.7	12.64 ± 0.17	16.59	—	—

^†^DF: days to flowering (days); PH: plant height (cm); NT: number of tillers; NP: number of panicles; YLD: yield (kg ha − 1); TGW: thousand grain weight (g); GL: grain length (mm); GW: grain width (mm); Fe: Iron (ppm); and Zn: Zinc (ppm).

### Identification of QTLs

**Figure 3:**
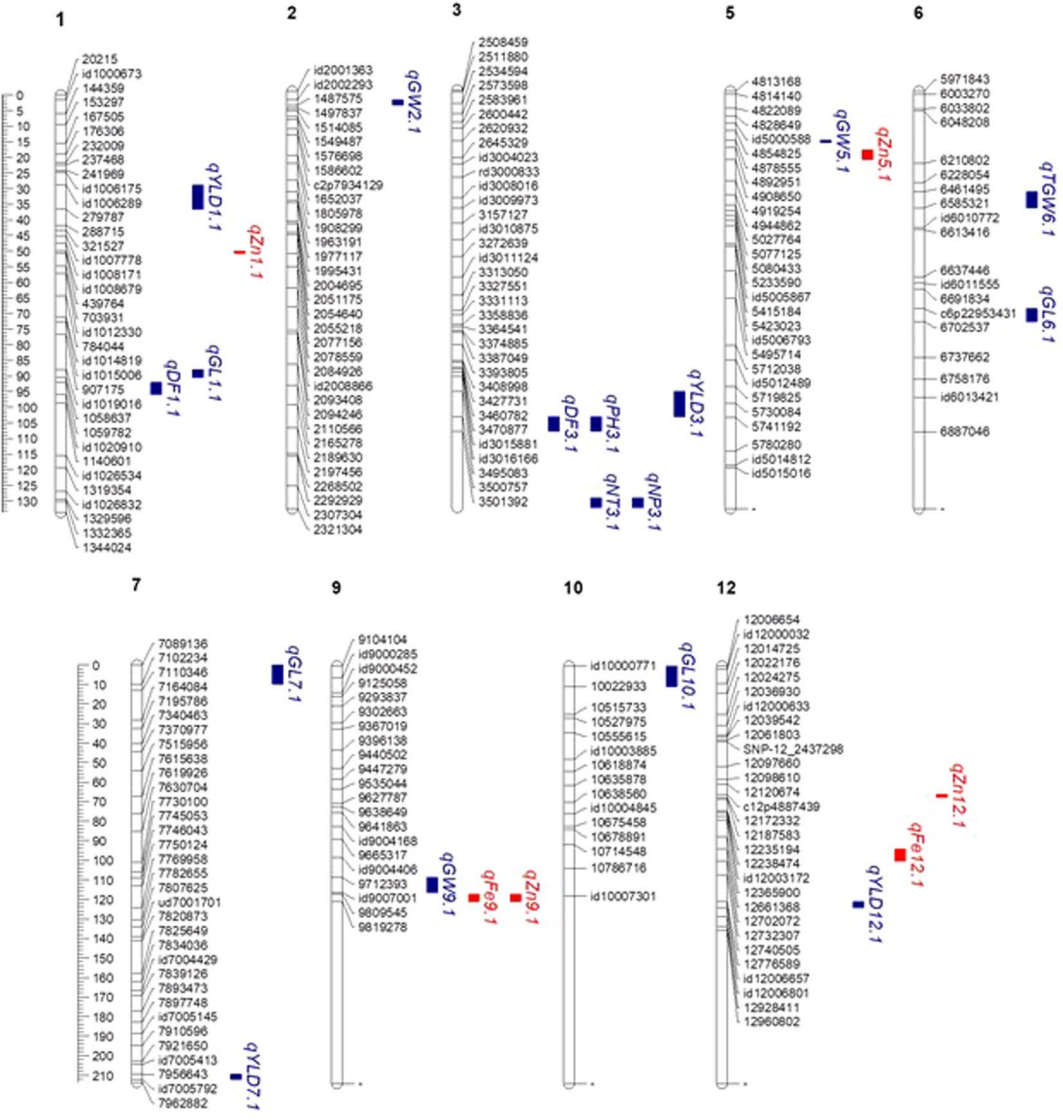
QTLs identified for agronomic traits and grain micronutrient concentrations in DH population. †Blue font: QTLs for agronomic traits; red font: QTLs for grain element concentrations.

A total of 23 QTLs for agronomic traits and grain Fe and Zn concentrations were identified in the DH population based on BLUEs (Table 2, Fig. 3). They were distributed on chromosomes 1, 2, 3, 5, 6, 7, 9, 10, and 12. The phenotypic variance explained (PVE) by the QTLs ranged from 7.18% to 22.03%. Two QTLs for DF (*qDF*_*1.1*_ and *qDF*_*3.1*_) were identified on chromosomes 1 and 3. These QTLs explained 14.82% and 12.63% PV. QTL *qDF*_*3.1*_ was contributed by the high-yielding parent IR05F102. One QTL each was identified on chromosome 3 for PH, NT and NP, each explained more than 12% PV. Four QTLs for YLD (*qYLD*_*1.1*_, *qYLD*_*3.1*,_ *qYLD*_*7.1*_ and *qDF*_*12.1*_) were identified on chromosomes 1, 3, 7, and 12 contributed by IR05F102. Three of them had PVE values of more than 10%. *qYLD*_*3.1*_ had the highest PVE (22.03%). One QTL for TGW was identified on chromosome 6 (*qTGW*_*6.1*_) contributed by IR05F102 with a PVE of 15.79 %. Similarly, four QTLs were identified for GL, one each on chromosomes 1, 6, 7 and 10. The PVE of these QTLs varied from 7.35% to 16.45%. *qGL*_*1.1*_, *qGL*_*6.1*_, and *qGL*_*7.1*_ had a PVE of more than 10% each. *qGL*_*6.1*_ had the highest PVE (16.45%). While, three QTLs for GW (*qGW*_*2.1*_, *qGW*_*5.1*_, and *qGW*_*9.1*_) were identified on chromosomes 2, 5, and 9 all derived from IR69428 with PVE values of 11.71%, 10.31%, and 14.76%, respectively. Two QTLs for Fe (*qFe*_*9.1*_ and *qFe*_*12.1*_) were identified on chromosomes 9 and 12 derived from IR69428 with PVE values of 11.79% and 13.34%, respectively. But for Zn, four QTLs were identified on chromosomes 1, 5, 9, and 12 contributed by IR69428. Three (*qZn*_*5.1*_, *qZn*_*9.1*_, *qZn*_*12.1*_) of them had PVE values of more than 10%. *qZn*_*12.1*_ had the highest PVE (15.26%).

**Table 2:** QTLs for agronomic traits and grain element concentrations in DH population.

Trait^†^	QTL	Chr	Marker Interval	POS (Mb)	LOD	PVE (%)	Add	Allele
DF (days)	*qDF*_*1.1*_	1	1059782–id1020910	32.14–33.67	8.00	14.82	−3.46	P2
	*qDF*_*3.1*_	3	3500757–3501392	33.97–33.98	6.70	12.63	2.63	P1
PH (cm)	*qPH*_*3.1*_	3	3500757–3501392	33.97–33.98	3.82	12.00	3.25	P1
NT	*qNT*_*3.1*_	3	id3017089–3536043	34.72–35.23	5.46	15.76	0.76	P1
NP	*qNP*_*3.1*_	3	id3017089–3536043	34.72–35.23	5.66	16.35	0.76	P1
YLD (kg ha^−1^)	*qYLD*_*1.1*_	1	id1006289–279787	8.17–9.22	5.39	11.91	501.63	P1
	*qYLD*_*3.1*_	3	3495083–3500757	33.71–33.97	9.36	22.03	499.34	P1
	*qYLD*_*7.1*_	7	7956643–id7005792	28.64–28.83	3.37	7.18	340.72	P1
	*qYLD*_*12.1*_	12	12776589–id12006657	18.59–19.78	4.95	10.95	400.64	P1
TGW (g)	*qTGW*_*6.1*_	6	6461495–6585321	17.27–20.14	4.61	15.79	0.90	P1
GL (mm)	*qGL*_*1.1*_	1	id1019016–1058637	31.52–32.09	6.05	14.41	0.31	P1
	*qGL*_*6.1*_	6	c6p22953431–6702537	22.95–22.96	6.59	16.45	0.25	P1
	*qGL*_*7.1*_	7	7089136–7102234	5.00–5.44	5.55	12.94	0.22	P1
	*qGL*_*10.1*_	10	id10000771–10022933	2.66–2.79	3.27	7.35	0.23	P1
GW (mm)	*qGW*_*2.1*_	2	id2002293–1487575	4.36–4.75	3.38	11.71	−0.02	P2
	*qGW*_*5.1*_	5	4878555–4884069	2.26–2.43	3.06	10.31	−0.02	P2
	*qGW*_*9.1*_	9	9712393–id9007001	16.53–19.66	4.24	14.76	−0.03	P2
Fe (ppm)	*qFe*_*9.1*_	9	9809545–9819278	19.79–20.13	3.36	11.79	−0.25	P2
	*qFe*_*12.1*_	12	12702072–12732307	16.98–17.57	3.98	13.34	−0.20	P2
Zn (ppm)	*qZn*_*1.1*_	1	id1008679–439764	12.71–13.79	3.14	8.96	−0.79	P2
	*qZn*_*5.1*_	5	4904312–4908650	3.26–3.42	4.13	12.15	−0.84	P2
	*qZn*_*9.1*_	9	9809545–9819278	19.79–20.13	4.56	13.79	−0.96	P2
	*qZn*_*12.1*_	12	c12p4887439–12172332	4.88–5.37	5.20	15.26	−0.77	P2

^†^DF: days to flowering (days); PH: plant height (cm); NT: number of tillers; NP: number of panicles; YLD: yield (kg ha-1); TGW: thousand grain weight (g); GL: grain length (mm); GW: grain width (mm); Fe: Iron (ppm); and Zn: Zinc (ppm).

### QTL co-locations

Three QTL co-locations consisting of QTLs for two traits each were observed on chromosomes 3 and 9, two for agronomic traits and one for grain mineral elements (Fig. 3). Interestingly, these traits that share common QTLs were positively correlated. Agronomic trait QTLs: *qDF*_*3.1*_ and *qPH*_*3.1*_ were co-located on chromosome 3 at 33.97–33.98 Mb within SNP marker interval 3500757–3501392 with allelic contribution from P1. QTLs *qNT*_*3.1*_ and *qNP*_*3.1*_ were co-located on chromosome 3 at 34.72–35.23 Mb within SNP marker interval id3017089 - 3536043 and with allelic contribution from P1. On the other hand, grain mineral element QTLs, *qFe*_*9.1*_ and *qZn*_*9.1*_ were co-located on chromosome 9 at 19.79–20.13 Mb within SNP marker interval id9809545 - 9819278 Mb with allelic contribution from P2.

### QTL x environment interaction analysis

**Figure 4:**
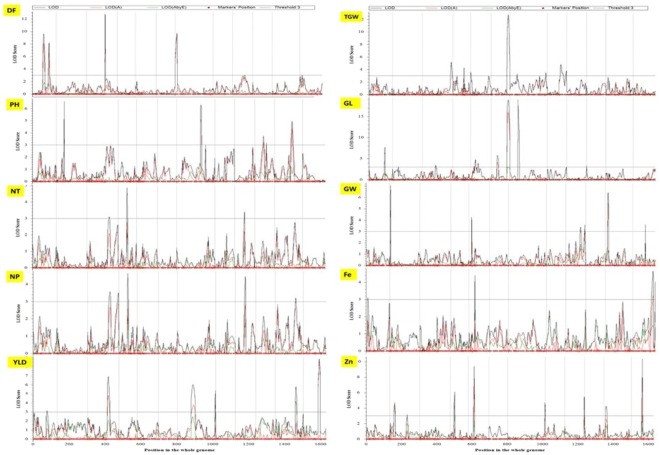
QTL by Environment Mapping: Chromosome locations of QTLs identified in the DH population.

To understand the stability and interaction of the identified QTLs with environment, QEI analysis was conducted. Estimated effects of 10 consistent QEI QTLs can be found in Table 3. One consistent QEI QTL was detected for DF on chromosome 1; two for YLD on chromosomes 3 and 7; two for GL on chromosomes 1 and 10; three for GW on chromosomes 3, 5 and 9; and two for Zn on chromosomes 9 and 12 (Fig. 4). All identified QEI QTLs were stable as indicated by the larger LOD_A_ (1.55–9.67) than LOD_AE_ (0.12–3.83) values and larger PVE_A_ (1.93–13.91%) than PVE_AE_ (0.05–3.21%) values (Table 3).

**Table 4:** Candidate genes for agronomic traits and grain Fe and Zn concentrations identified in the DH population.

QTL	Gene names	Locus names	Location	GO/TO	Reference
*qDF*_*1.1*_	*OsLFL1*	*Os01g0713600*	2.49 Mb left of QTL	flower development trait	(Peng *et al*., 2008)
*qDF*_*3.1*_	*OsHD6*	*Os03g0762000*	2.46 Mb left of QTL	days to heading	(Takahashi *et al*., 2001)
*qPH*_*3.1*_	*OsLTS1*	*Os03g0837300*	1.20 Mb right of QTL	plant height	
*qYLD*_*3.1*_	*OsDST*	*Os03g0786400*	Within QTL	grain number	
	*OsIPT4*	*Os03g0810100*	Within QTL	Grain number	(Liu *et al*., 2011)
*qYLD*_*7.1*_	*OsMED5_3*	*Os07g0681500*	0.05 Mb right of QTL	1000-seed weight/grain width/grain yield	(Malik *et al*., 2016)
*qYLD*_*12.1*_	*OsNAC139*	*Os12g0477400*	1.20 Mb left of QTL		
*qGW*_*5.1*_	*OsSRS3*	*Os05g0154700*	0.76 Mb right of QTL	grain size	(Kitagawa *et al*., 2010)
	*OsGS5, OsSCP26*	*Os05g0158500*	1.00 Mb right of QTL	grain shape	(Li *et al*., 2011)
*qFe*_*9.1*_	*OsLysM-RLK10, OsRLCK276*	*Os09g0511000*	Within QTL	2Fe-2S cluster binding; electron transporter	
*qFe*_*12.1*_	*SWEET13, OsSWEET13*	*Os12g0476200*	Within QTL	haem binding	
*qZn*_*1.1*_	*OsGATA8, OsGATA14*	*Os01g0343300*	Within QTL	zinc binding	
	*OsARL1e, ARL1e, Sar1b*	*Os01g0338000*	Within QTL	zinc binding	
*qZn*_*5.1*_	*OsZIP6*	*Os05g0164800*	0.38 Mb right of QTL	rice ferrous ion transporter	(Ishimaru *et al*., 2005)
*qZn*_*9.1*_		*Os09g0511500*	Within QTL	zinc binding

### Epistatic interaction analysis

One epistatic interaction for PH was identified in the DH population (Supplementary Table 4; Fig. S2). The major di-genic epistatic interaction was identified between loci on chromosomes 3 with SNP marker interval id3495083 - 3500757 Mb and on chromosome 7 with SNP marker interval id7862147 - 7892971 Mb with a PVE value of 28.40%.

### Candidate genes underlying QTLs for agronomic traits, Fe and Zn

Seven agronomic trait QTLs and five Fe and Zn QTLs were harbored in or near candidate genes (Table 4). For agronomic traits, two candidate genes (*OsLFL1* and *OsHD6*) were identified for DF located on chromosomes 1 and 3. *OsLFL1* with locus id *Os01g0713600* was identified as a gene for flower development while *OsHD6* with locus id *Os03g0762000* was for days to heading. One candidate gene, *OsLTS1* was identified for PH on chromosome 3 with locus id *Os03g0837300*. Four candidate genes were identified for YLD such as, *OsDST, OsIPT4, OsMED5_3*, and *OsNAC139* on chromosomes 3, 7, and 12, respectively. Two candidate genes for GW, *OsSRS3* and *OsGS5* were identified on chromosome 5. Among QTLs for grain mineral element concentrations, for Fe, *OsLysM-RLK10* and *OsSWEET13* were candidate genes identified on chromosome 9 and 12, while candidate genes *OsGATA8*, *OsSar1b, OsZIP6*, and *Os09g0511500* on chromosomes 1, 5, and 9 were identified for Zn (Supplementary Figs. S3 and S4). There were significant sequence differences in the candidate genic regions of the major QTLs (Supplementary Table S5).

**Table 3:** Estimated effects of QTL by Environment Interaction QTLs of agronomic traits and grain element concentrations identified in the DH population.

Trait	QTL	Chr	Marker Interval	LOD	LOD	LOD	PVE(%)	PVE (%)	PVE (%)	Additive effect			Average
					(A)	(AE)		(A)	(AE)	2016DS	2017DS	2017WS	effect
DF	*qDF*_*1.1*_	1	1059782–id1020910	8.13	6.51	1.62	7.41	5.9	1.52	1.24	−1.21	−0.04	−1.97
YLD	*qYLD*_*3.1*_	3	3495083–3500757	6.93	4.84	2.09	7.87	6.12	1.75	−60.78	266.09	−205.31	368.77
	*qYLD*_*7.1*_	7	id7005792–7962882	5.38	1.55	3.83	2.93	1.93	1	−240.07	41.51	198.55	252.56
GL	*qGL*_*1.1*_	1	1058637–1059782	7.69	6.55	1.13	6.54	5.35	1.18	−0.07	0.14	−0.07	0.22
	*qGL*_*10.1*_	10	id10000771–10022933	3.33	3.2	0.12	2.62	2.53	0.09	0.04	−0.01	−0.03	0.15
GW	*qGW*_*2.1*_	2	id2002293–1487575	7.07	4.86	2.21	10.21	7.78	2.43	−0.01	0.02	−0.01	−0.03
	*qGW*_*5.1*_	5	4878555–4884069	4.26	2	2.25	5.4	3.09	2.31	−0.02	0.01	0.01	−0.02
	*qGW*_*9.1*_	9	9712393–id9007001	3.58	3.3	0.28	5.04	4.98	0.05	0	0	0	−0.02
Zn	*qZn*_*9.1*_	9	9809545–9819278	5.48	2.61	2.87	7.09	3.88	3.21	0.05	−0.6	0.55	−0.51
	*qZn*_*12.1*_	12	c12p4887439–12172332	10.33	9.67	0.66	16.93	13.91	3.02	−0.38	−0.11	0.49	−0.78

### QTL classes for YLD and Zn

To understand the effects of combining the identified QTLs for YLD and Zn in this study, QTL classes were calculated. The mean YLD of DH lines without QTLs was 4430.9 kg ha^−1^. On the other hand, the mean Zn of the DH lines without QTLs was 11.18 ppm. The genotype classes for YLD and Zn showed a wide range of 4129.4–6373.0 kg ha^−1^ and 11.1–17.7 ppm, respectively. An increase in YLD and Zn was observed with an increase in the number of QTLs (Tables 5 and S3). The IR05F102 allele at all four loci increased YLD. DH lines with three to four QTLs (genotype class 15 and 16) showed the highest YLD, 6373.0 kg ha^−1^ and 6285.7 kg ha^−1^, respectively, among all YLD genotype classes. The IR69428 alleles at all four loci increased Zn. DH lines with four QTLs (*qZn*_*1.1*_ + *qZn*_*5.1*_ + *qZn*_*9.1*_ + *qZn*_*12.1*_) showed the highest Zn, 17.7 ppm, among all Zn genotype classes.

**Table 5:** Means and effects of 12 QTL classes for Zn across three seasons.

SN	QTL class	Mean (ppm)	Effect^a^ (ppm)
1	*None*	11.18^b^	0.00
2	*qZn*_*1.1*_	11.10^b^	−0.08
3	*qZn*_*5.1*_	12.43^a,b^	1.25
4	*qZn*_*9.1*_	11.84^a,b^	0.66
5	*qZn*_*12.1*_	13.05^a,b^	1.87
6	*qZn*_*1.1*_ + *qZn*_*5.1*_	13.78^a,b^	2.60
7	*qZn*_*1.1*_ + *qZn*_*12.1*_	13.47^a,b^	2.29
8	*qZn*_*5.1*_ + *qZn*_*9.1*_	12.36^a,b^	1.18
9	*qZn*_*5.1*_ + *qZn*_*12.1*_	14.74^a,b^	3.56
10	*qZn*_*1.1*_ + *qZn*_*9.1*_ + *qZn*_*12.1*_	15.25^a,b^	4.07
11	*qZn*_*5.1*_ + *qZn*_*9.1*_ + *qZn*_*12.1*_	15.73^a,b^	4.55
12	*qZn*_*1.1*_ + *qZn*_*5.1*_ + *qZn*_*9.1*_ + *qZn*_*12.1*_	17.70^a^	6.52

^a^Difference to the class with no QTLs.

^b^Different letters indicate significant differences by Tukeys’s Honest Significant Difference (HSD) Test multiple comparison of means by P < 0.05.

### Identification of DH lines with good agronomic traits and high grain Zn

The top ten high Zn lines were identified using BLUEs of the 148 DH lines (Table 6). IR 91153-AC 113-1 was early for DF and had the highest YLD (6697.23 kg ha^−1^) among the top lines. All four YLD QTLs (*qYLD*_*1.1*_, *qYLD*_*3.1*_, *qYLD*_*7.1*_, *qYLD*_*12.1*_) and one Zn QTL (*qZn*_*12.1*_) were detected in this line. IR 91153-AC726-1 had the highest value for Zn and Fe of 16.21 and 4.82 ppm, respectively. Two Zn QTLs were identified in this line (*qZn*_*5.1*_, *qZn*_*12.1*_) and one YLD QTL (*qYLD*_*1.1*_). These DH lines can be used as donors in breeding programs or can be directly tested in multi-location trials to further evaluate their performance and potential to be released as high Zn rice varieties.

**Table 6:** Agronomic traits and Fe and Zn concentrations of the parents and top ten high-Zn rice DH lines.

Designation	DF (days)	PH (cm)	YLD (kg ha^−1^)	Fe (ppm)	Zn (ppm)	QTLs
IR 91153-AC 113-1	88.25	98.13	6697.23	4.26	15.11	*qZn*_*12.1*_*, qYLD*_*1.1*_*, qYLD*_*3.1*_*, qYLD*_*7.1*_*, qYLD*_*12.1*_
IR 91153-AC 325-1	88.61	91.09	5900.48	3.29	14.13	*qYLD*_*1.1*_*, qYLD*_*3.1*_*, qYLD*_*7.1*_*, qYLD*_*12.1*_
IR 91153-AC 374-2	80.30*	94.71	5150.38	4.05	13.85	*qYLD*_*1.1*_*, qYLD*_*7.1*_
IR 91153-AC 545-1	89.84	97.58	5648.43	4.09	14.47	*qZn*_*9.1*_*, qYLD*_*3.1*_*, qYLD*_*12.1*_
IR 91153-AC 568-7	83.05*	92.03	6092.35	3.69	14.13	*qYLD*_*1.1*_*, qYLD*_*3.1*_
IR 91153-AC 579-1	92.23	95.87	5942.92	4.71-	13.84	*qYLD*_*1.1*_*, qYLD*_*7.1*_*, qYLD*_*12.1*_
IR 91153-AC 701-1	93.92	98.89	5160.19	4.27	14.42	*qYLD*_*1.1*_*, qYLD*_*7.1*_*, qYLD*_*12.1*_
IR 91153-AC 701-2	92.71	92.24	5689.03	4.28	13.88	*qYLD*_*1.1*_*, qYLD*_*3.1*_*, qYLD*_*12.1*_
IR 91153-AC 722-1	93.72	88.69	5944.21	3.76	14.03	*qYLD*_*1.1*_*, qYLD*_*3.1*_*, qYLD*_*12.1*_
IR 91153-AC 726-1	86.76	95.63	5006.42	4.82-	16.21	*qZn*_*5.1*_*, qZn*_*12.1*_*, qYLD*_*1.1*_
IR05F102	88.50	95.40	6425.10	3.60	3.90	*qYLD*_*1.1*_*, qYLD*_*3.1*_*, qYLD*_*7.1*_*, qYLD*_*12.1*_
IR69428	94.50	98.90	3154.40	11.90	14.80	*qZn*_*1.1*_*, qZn*_*5.1*_*, qZn*_*9.1*,_ *qZn*_*12.1*_

^§^DF: days to flowering (days); PH: plant height (cm); YLD: yield (kg ha-1); Fe: Iron (ppm); and Zn: Zinc (ppm).

## Discussion

Compared to other cereals, rice is a poor source of essential micronutrients to fulfill daily human nutritional requirements^33^. Zn biofortification is considered to be a major solution that appears to be the most sustainable and cost-effective approach for addressing micronutrient malnutrition or hidden hunger^34^. Knowledge of genetic variation in agronomic traits, grain mineral elements concentration such as Fe and Zn and genes underlying allelic variation is vital for rice biofortification breeding to fast track the development of rice varieties that can make a positive contribution to human health. In this study, a DH population of rice was used to map QTLs for agronomic traits, Fe and Zn concentrations with SNP markers and BLUEs. Epistatic interactions, QTL by environment interactions, QTL pyramiding effects and haplotype analysis of major QTLs for YLD and Zn were also examined.

The DH population showed wide variation in all traits studied which specifies the traits complex and polygenic nature. BLUEs for grain Fe showed relatively little variation while grain Zn ranged from 8.7 to 19.7 ppm. The variation observed in the DH population for agronomic traits and grain Zn is important and a prerequisite for QTL mapping^35^. The variation indicates that a proportion of the phenotypic variance can be attributed to genotypic variance and that the agronomic traits and grain Zn can be exploited through selective breeding^36^. A wide range of variation also displayed the role of genotype as well as the effects of environment on the expression of the studied traits^37^. Several prior studies have also reported significant genetic variation specifically for grain Zn concentration^38–40^ which supports that selection for high levels of Zn coupled with advanced molecular marker technologies is a feasible approach.

High H^2^ values (>0.50) for all agronomic traits and grain Zn concentration were observed in the DH population which is consistent with previous studies that also work with rice agronomic traits and grain micronutrient concentrations^41–43^. However, the heritability was much lower for grain Fe concentration. Reliability of early generation selection of highly heritable agronomic traits (DF, PH, TGW, GW, and GL) is highly feasible, which may result in significant response to selection^44^. The low heritability observed for Fe indicates significant influence of environment and early selection for Fe is not feasible.

The first two PCs explained 55.94% of the total variation observed in the DH population. In general, principal components with vector coefficients more than 0.3 irrespective of their direction of influence are considered important^45^. Component loadings for each principal component revealed agronomic traits, such as YLD, TGW, GL and GW were among the phenotypic traits contributing positive projections while NT, NP, Fe and Zn contributing negative projections in two principal components which explained 30.34% and 25.60% of variation respectively. Through PCA we identified a number of traits which are responsible for the observed genotypic variation in the DH population, thereby identifying the traits with greatest impact on the phenotype of DH lines, which is informative for the selection of lines in breeding programs.

Analysis of relationships among quantitative traits is important for assessing the feasibility of joint selection of two or more traits and the effect of selection for secondary traits in genetic gain for the primary trait^46^. A positive genetic correlation between two desirable traits makes the job of the plant breeder easy in improving both traits simultaneously^47^. Clear insight on genetic correlations of agronomic traits and grain Fe and Zn concentrations can help plant breeder devise a suitable breeding strategy to enhance micronutrient density in rice. In the present study, DF was negatively correlated with TGW in BLUEs and across seasons, while PH was positively correlated with YLD. The trait NT was positively correlated with NP and YLD, but negatively correlated with GW. Similarly, GL was negatively correlated with Fe, while Fe and Zn were positively correlated. Previous studies also reported a positive correlation between grain Fe and Zn concentrations in rice^15,16,39,40^ which infers common mechanisms for uptake, translocation and loading of Fe and Zn, and suggests that grain Zn can also be used for grain Fe selection. Negative correlations between YLD and Zn have also been observed^39,40^, though it was not consistent, and these highlight the need for biofortification breeding programs to give significant weight to YLD while selecting for high grain Zn genotypes.

Considering the agronomic traits such as DF, PH, and YLD, Fe and Zn, the present study identified a DH line for which YLD was 6697.23 kg ha^−1^ with a Zn content of 15.11 ppm (IR 91153-AC 113-1). The high Zn lines identified in this study could be used in biofortification breeding programs to improve the grain Zn levels in rice resulting in improved rice varieties that could directly impact human nutrition, especially in populations that heavily rely on rice-based diets.

In this study, seventeen QTLs were mapped for agronomic traits and six QTLs were identified for Fe and Zn. It was notable that all the QTLs for grain Fe and Zn concentrations as well as QTLs for GW were derived from IR69428. Meanwhile, IR05F102 contributed all the QTLs for agronomic traits PH, NP, NT, YLD, TGW, and GL. These QTLs are good candidates for further studies such as gene fine-mapping and cloning. Our QTL results corroborated with recent studies on QTL mapping for agronomic traits and grain Fe and Zn concentrations in rice using diverse populations such as RILs^26^, BILs^28^, BC_2_F_3_ derived lines^19^, DH lines^15^, and MAGIC lines^40^ that revealed multiple loci located on all rice chromosomes. Consequently, these QTLs were detected in different environments and genetic backgrounds. The above results clearly showed the complexity of agronomic traits and grain Zn concentration^14,15,42^. Since the present study used BLUE values which excluded environmental effects and magnify genetic effects, the QTLs identified in this study may well prove more useful in rice biofortification breeding programs than the QTLs identified using single environment phenotypic values.

**Figure 5:**
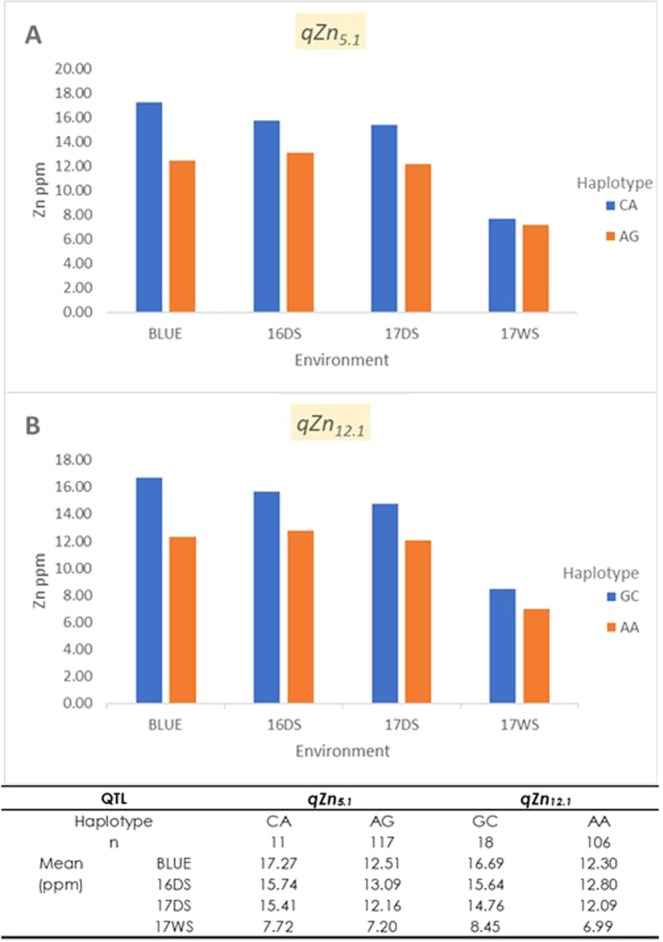
The haplotypes of the major effect QTLs for grain Zn, *qZn*_*5.1*_ (**A**) and *qZn*_*12.1*_ (**B**), and their effect on Zn phenotype in different environments. Blue and orange color indicates different haplotypes within the QTLs, n indicates the number of DH lines with different haplotypes.

Haplotypes for two stable and major effect QTLs for grain Zn were further examined (Fig. 5). For *qZn*_*5.1*_, two haplotypes, CA and AG were observed in the DH population indicating that there is a tight linkage between the two loci. The CA haplotype is associated with high Zn. Similarly, the two haplotypes in *qZn*_*12.1*_ were GC and AA. The latter was associated with high Zn. The stability of the markers in these loci has clearly shown its potential for use in biofortification breeding programs although further validation is still necessary.

Co-localized QTLs are important for simultaneous improvement of traits of interest such as grain Fe and Zn concentrations in rice from plant breeder point of view^48^. QTLs controlling correlated traits were usually mapped in the same or close chromosome regions^49^. Some previous studies have reported the phenomenon of QTL co-locations for rice micronutrient concentrations^15,19,23^. In this study, several co-located QTLs linked with correlated traits were identified on chromosomes 3 and 9. For instance in agronomic traits, *qDF*_*3.1*_ and *qPH*_*3.1*_ were co-localized on chromosome 3 in the same SNP marker interval 3500757–3501392. For grain Fe and Zn, *qFe*_*9.1*_ and *qZn*_*9.1*_ were co-localized in the same SNP marker interval 9809545–9819278. These co-located QTLs may be the result of pleiotropy or multiple tight linkage of genes controlling the traits^50^. These regions might be useful for development of high grain Zn rice with highly productive agronomic traits. The co-location of QTLs for Fe and Zn will be beneficial for their simultaneous improvement with Marker Assisted Breeding^15,19,23^.

Identification of environment-specific and stable QTLs having consistent genetic effects across a wide range of environments is of great importance in rice biofortification breeding^37,40^. Several QTLs for agronomic traits and grain Fe and Zn concentrations have been identified, but the positions, effect sizes and genetic effect directions of these QTLs were mostly genotype and environment specific^51^. Studies on QEI contribute to the effective use of marker-assisted selection (MAS) in biofortification breeding, better understanding of the genetic architecture of grain Zn and agronomic traits, and the interactions of the genotype and environment^52^. The QTLs that were only detected in one environment mostly showed poor stability. All the consistent QTLs identified in this study: one for DF; two for YLD, GL, and Zn; and three for GW based on QEI analysis confirms stability as indicated by higher LOD_A_ and PVE_A_ values as compared to LOD_AE_ and PVE_AE_ values. The environment plays only a minor role in the additive effects of these QTLs. For instance, the additive effects of Zn QTL *qZn*_*12.1*_ were 0.38 in S1, whereas in S2 the additive effects were only 0.11.

Expression of the phenotype is a result of polygenes interacting to control complex traits^53^. Understanding epistatic interactions can be useful to target genes through marker-assisted selection strategies for the improvement of complex traits such as YLD and grain Zn. In this study, we detected one epistatic interaction on chromosomes 3 and 7 for PH. The total variation explained by epistatic loci for PH were significant suggesting that epistasis in the form of additive by additive interactions, plays a very important role in controlling PH.

Several QTLs harbor genes for agronomic traits and grain Fe and Zn concentration. In this study, genes for flowering development on the chromosome 1 and days to heading on chromosome 3 were co-located with QTLs identified for DF^54,55^. *OsDST*, *OsIPT4*, *OsMED5_3*, and *OsNAC139* genes for grain number, 1000-seed weight, and grain width^56^ co-located with QTLs identified for YLD on chromosomes 3, 7, and 12. The candidate genes for grain Fe and Zn concentrations are involved in uptake, translocation, and metal homeostasis within rice plants. A network analysis of genes *Sar1a* and *SWEET13* were conducted to understand their interaction with other traits and genes through the Knetminer program (Supplementary Figs. S3 and S4). *Sar1a* was positioned within 12.71–13.79 Mb on chromosome 1 for Zn while *SWEET13* was positioned within 16.98–17.57 Mb on chromosome 12 for Fe. Six QTLs and 35 traits were linked to *Sar1a*, while 12 QTLs and 42 traits were linked to *SWEET13*. A QTL for Zn on chromosome 5 was linked to candidate gene *OsZIP6*, which translocates Fe and Zn and may be responsible for high concentrations of these micronutrients in the grains^57,58^ These candidate genes which were tightly linked with QTLs identified are worthy of further investigation.

QTL pyramiding is successfully utilized in rice breeding to develop lines resistance/tolerance to biotic and abiotic stresses^59,60^. To evaluate the pyramiding effect of each QTL in the present study, we compared the means of YLD and Zn between genotype classes in all combinations. Although, the arithmetic mean of YLD of all the genotype classes were higher than that of class 1 (no QTLs), no significant differences were detected. However, the means of class 15 (6373.00 kg ha^−1^) and class 16 (6285.74 kg ha^−1^) were significantly higher than that of class 4 (4129.43 kg ha^−1^). On the other hand, the mean Zn concentration of classes 3 to 11 ranged from 11.84 to 15.73 ppm, which was higher than that of class 0, though there were no significant differences detected among these groups. A significant difference was detected between class 12 (17.70 ppm) and class 1 (11.18 ppm). Hence, it will be necessary to pyramid favorable alleles of Zn and YLD QTLs in popular variety background to accumulate a large number of desirable alleles.

## Conclusion

The DH population showed wide variation for agronomic traits, grain Fe and Zn. High heritability was obtained for DF, PH, and GL and moderate for TGW and Zn. Significant positive correlations between PH & YLD, YLD & GL, TGW & GW, and Fe & Zn were observed. Most of the QTL alleles associated with improved grain Zn and Fe were contributed by the donor parent IR69428. Six QTLs contributed more than 15% of the phenotypic variance; *qNT*_*3.1*,_ *qNP*_*3.1*,_ *qYLD*_*3.1*,_ *qTGW6*_*.1*,_ *qGL*_*6.1*,_ and *qZn12*_*.1*._ Based on QEI analysis, all the consistent QTLs were relatively stable. The epistatic QTL analysis detected one significant di-genic interaction for PH with 28.40% PVE. QTLs for grain Fe and Zn concentrations were associated with the candidate genes invovlved in metal homeostasis. A positive correlation between YLD and Zn levels was found in several combinations of three to four QTLs. The high Zn lines identified in this study could be used in biofortification breeding program to improve the grain Zn levels in rice resulting in improved rice varieties that could directly impact human nutrition.

## Methods

### Plant materials

A doubled-haploid (DH) population, derived from IR05F102 (P1) x IR69428 (P2) cross composed of 148 lines, was used in the study. The mapping population was evaluated under irrigated conditions during the dry seasons of 2016 and 2017 (S1, S2) and wet season of 2017 (S3) in Robert Zeigler Experimental Station (ZES) at International Rice Research Institute (IRRI), Los Baños (LB), Laguna. The experiment was laid out in Randomized Complete Block Design (RCBD) with two to three replications per trial. Seedlings were transplanted at 21 days with a spacing of 20 × 20 cm, and the plant population was 2 rows of 10 hills in each plot. Standard agronomic practices and plant protection measures were applied to ensure good crop growth and complete grain development.

### Phenotyping

The population was evaluated for eight agronomic traits and two grain mineral elements. The agronomic traits were measured following the standard evaluation system^61^, including days to 50% flowering (DF), plant height (PH), number of tillers (NT), number of panicles (NP), grain yield (YLD), thousand grain weight (TGW), grain length (GL), and grain width (GW). For grain Fe and Zn analysis, 50 g paddy samples were dehulled using a Satake dehuller and milled for one minute using a K-710 mini-lab rice polisher. Milled rice samples weighing at least 3 g representing each plot were analyzed using X-ray Fluorescence Spectrometry (XRF) (Oxford)^62^. Measurements were done twice per sample and was expressed in parts per million (ppm). The average reading per plot was considered for further statistical analysis.

### Statistical analysis

A summary of basic statistical parameters was generated using STAR v.2.0.1. Analysis of Variance (ANOVA) was performed on single-season data and combined season data using one-stage multi-environment analysis implemented in PBTools v1.4. Best linear unbiased estimates (BLUEs) were generated by setting genotype effects as fixed and season effects as random. The BLUEs were used to perform QTL analysis. Boxplots and Pearson^,s^ correlation coefficients between pairs of traits were estimated using R Core team^63^.

The model used for ANOVA was:

$${{Y}}_{{ijko}}={\mu }+{{\alpha }}_{{i}}+{{r}}_{{j}}+{{l}}_{{kj}}+{{b}}_{{ojk}}+{{\varepsilon }}_{{ijko}}$$

where *μ* is the overall mean, *α*_*i*_ is the effect of the *i*^*th*^ genotype; *r*_*j*_ is the effect of the *j*^*th*^ season, *l*_*kj*_ the effect of the *k*^*th*^ replicate within the *j*^*th*^ season, *b*_*ojk*_ was the effect of the *o*^*th*^ block at the *j*^*th*^ season of the *k*^*th*^ replicate and *ε*_*ijk*_ the error. The genotypes were considered fixed while replicates and blocks within replicates were random.

Broad-sense heritability (H^2^) for each trait in each season was calculated as:

$${{H}}^{{2}}=\frac{{{\sigma }}_{{g}}^{{2}}}{{{\sigma }}_{{p}}^{{2}}}\,and\,{{\sigma }}_{{p}}^{{2}}={{\sigma }}_{{g}}^{{2}}+\frac{{{\sigma }}_{{e}}^{{2}}}{{r}}$$

where ${{\sigma }}_{{p}}^{{2}}$ is the phenotypic variance, ${{\sigma }}_{{g}}^{{2}}$ is the genotypic variance, ${{\sigma }}_{{e}}^{{2}}$ is the error variance, and r is the number of replications.

### Genotyping

Plant genomic DNAs were extracted from leaf tissue using the cetyl trimethylammonium bromide (CTAB) method^64^. Quality check of DNA samples was carried out in 1% agarose gel. We submitted ~50 ng of DNA from the complete set of the doubled-haploid mapping population, along with the parents for genotyping with 7 K SNP array technology at the Genotyping Services Laboratory (GSL) at IRRI. Scanned image calls and automatic allele calling were loaded in the Illumina Genome Studio data analysis version V2011.1.

### Linkage mapping and QTL analysis

The linkage map of the DH population was constructed using 379 high quality SNP markers using IciMapping v4.1^65^. The distribution of SNP markers varied across chromosomes. The number of SNPs per chromosome ranged from 15 SNPs (chromosomes 8 and 9) to 57 SNPs (chromosome 3). The total length of the linkage map was 1,629.6 cM while the average interval length was 4.3 cM (Supplementary Fig. S5). The linkage map was created using Kosambi function^66^. For analysis of QTLs, the BLUEs of each line in the DH population were used. The permutation method was used to obtain an empirical threshold for claiming QTLs based on 1000 runs of randomly shuffling the trait values at 95% confidence level using the BIP function, whereas epistatic interactions were identified by setting the logarithm of odds (LOD) threshold value at 6.0 utilizing inclusive composite interval mapping (ICIM) model in QTL IciMapping ver.4.1. The MET function in ICIMapping was used to study QTL x Environment Interaction. QTLs were visualized using MapChart v.2.3^67^.

### Candidate gene analysis

The physical position of each QTL was determined by the position of the flanking SNP markers and genes physically located within or near QTLs for agronomic and grain Fe and Zn traits were considered candidate genes. Annotated genes with functions related to agronomic traits, metal transport and homeostasis were compiled and the physical positions of annotated genes were determined using the RAP DB Genome Browser^68^ (http://rapdb.dna.affrc.go.jp/viewer/gbrowse/ irgsp1). Annotation and functions attributed to different candidate genes were downloaded from Oryzabase^69^ (https://shigen.nig.ac.jp/rice/oryzabase/gene). A network of the top hit candidate genes and QTLs was created using K-netminer program^70^ (http://knetminer.rothamsted.ac.uk/Oryza_sativa/).

## Supplementary information

Supplementary information.
