# Supplementary material for: Genetic Analysis of Agronomic Traits and Grain Iron and Zinc Concentrations in a Doubled Haploid Population of Rice (Oryza sativa L.)
Source: Sci Rep. 2020 Feb 10;10:2283. doi: 10.1038/s41598-020-59184-z (PMC7010768; doi:10.1038/s41598-020-59184-z)
Supplement: Supplementary file 1 — Supplementary information. [file 41598_2020_59184_MOESM1_ESM.docx]

**Genetic Analysis of Agronomic Traits and Grain Iron and Zinc Concentrations in a Doubled Haploid Population of Rice (*Oryza sativa* L.)**

Mark Ian C. Calayugan, Gwen Iris Descalsota-Empleo, Chau Thanh Nha, Andrea Kariza Formantes, Mary Ann Inabangan-Asilo, Amery Amparado, Zin Mar Swe, Jose E. Hernandez, Teresita H. Borromeo, Antonio G. Lalusin, Merlyn S. Mendioro, Ma. Genaleen Q. Diaz, Celia B. dela Viña, Russell Reinke, and B.P. Mallikarjuna Swamy

Table S1. Pearson’s correlation coefficient between BLUE values and seasonal values for agronomic traits and grain Fe and Zn concentrations in rice DH population.

| **Trait** | **Pearson correlation** | | |
| --- | --- | --- | --- |
|  | **BLUEs –S1** | **BLUEs – S2** | **BLUEs – S3** |
| DF (days) | 0.87*** | 0.94*** | 0.97*** |
| PH (cm) | 0.90*** | 0.91*** | 0.83*** |
| NT | 0.58*** | 0.79*** | 0.76*** |
| NP | 0.62*** | 0.78*** | 0.75*** |
| YLD (kgha^-1^) | 0.52*** | 0.86*** | 0.87*** |
| TGW (g) | 0.88*** | 0.97*** | 0.93*** |
| GL (mm) | 0.97*** | 0.97*** | 0.95*** |
| GW (mm) | 0.92*** | 0.56*** | 0.76*** |
| Fe (ppm) | 0.87*** | 0.76*** | 0.56*** |
| Zn (ppm) | 0.89*** | 0.84*** | 0.79*** |

Table S2. Factor loadings (eigenvectors) for the agronomic traits and grain Fe and Zn concentrations, and computed eigenvalues for the first two principal components.

| **Variable**† | **PC1** | **PC2** |
| --- | --- | --- |
| Eigenvalue | 1.7418 | 1.6001 |
| Variability (%) | 0.3034 | 0.256 |
| Cumulative (%) | 0.3034 | 0.5594 |
|  |  |  |
| DF (days) | 0.01 | -0.18 |
| PH (cm) | 0.29 | 0.13 |
| NT | 0.01 | **-0.54** |
| NP | 0.03 | **-0.55** |
| YLD (kgha^-1^) | **0.44** | -0.18 |
| TGW (g) | 0.29 | **0.37** |
| GL (mm) | **0.46** | 0.11 |
| GW (mm) | -0.18 | **0.4** |
| Fe (ppm) | **-0.43** | -0.09 |
| Zn (ppm) | **-0.46** | 0.14 |

Table S3. Means and effects of 16 QTL classes for YLD in DH population across three seasons

| **SN** | **QTL class** | **Mean (kgha^-1^)** | **Effect^a^ (kgha^-1^)** |
| --- | --- | --- | --- |
| 1 | None | 4430.89 _ab_ | 0 |
| 2 | *qYLD_1.1_* | 4705.28 _ab_ | 274.39 |
| 3 | *qYLD_3.1_* | 5743.05 _ab_ | 1312.16 |
| 4 | *qYLD_7.1_* | 4129.43 _b_ | -301.46 |
| 5 | *qYLD_12.1_* | 4844.57 _ab_ | 413.68 |
| 6 | *qYLD_1.1_+qYLD_3.1_* | 5434.88 _ab_ | 1003.99 |
| 7 | *qYLD_1.1_+qYLD_7.1_* | 4641.82 _ab_ | 210.93 |
| 8 | *qYLD_1.1_+qYLD_12.1_* | 4633.73 _ab_ | 202.84 |
| 9 | *qYLD_3.1_+qYLD_7.1_* | 5174.09 _ab_ | 743.2 |
| 10 | *qYLD_3.1_+qYLD_12.1_* | 5370.39 _ab_ | 939.5 |
| 11 | *qYLD_7.1_+qYLD_12.1_* | 4861.09 _ab_ | 430.2 |
| 12 | *qYLD_1.1_+qYLD_3.1_+qYLD_7.1_* | 4928.28 _ab_ | 497.39 |
| 13 | *qYLD_1.1_+qYLD_3.1_+qYLD_12.1_* | 5714.47 _ab_ | 1283.58 |
| 14 | *qYLD_1.1_+qYLD_7.1_+qYLD_12.1_* | 5818.87 _ab_ | 1387.98 |
| 15 | *qYLD_3.1_+qYLD_7.1_+qYLD_12.1_* | 6373.00 _a_ | 1942.11 |
| 16 | *qYLD_1.1_+qYLD_3.1_+qYLD_7.1_+qYLD_12.1_* | 6285.74 _a_ | 1854.85 |

a Difference to the class with no QTLs

b Different letters indicate significant differences by Tukeys's Honest Significant Difference (HSD) Test multiple comparison of means by P<0.05

| **Trait** | **Chr** | **Marker Interval** | **Chr** | **Marker Interval** | **LOD** | **PVE (%)** | **Add1** | **Add2** | **Add x Add** |
| --- | --- | --- | --- | --- | --- | --- | --- | --- | --- |
| PH | 3 | 3495083- 3500757 | 7 | 7862147- 7892971 | 6.07 | 28.4 | 5.11 | 2.39 | 6.21 |

Table S4. Epistatic interactions identified in DH population

Table S5. Polymorphic SNP sequences within the candidate genes underlying major QTLs

| **QTL** | **Locus / Gene Names** | **CHROMOSOME** | **POSITION** | **NIPPONBARE** | **IR 05F101** | **IR69428** |
| --- | --- | --- | --- | --- | --- | --- |
| *qDF_1.1_* | *LOC_Os01g0713600_OsLFL1* | 1 | 29727014 | C | T | C |
|  |  | 1 | 29729594 | G | T | G |
|  |  | 1 | 29758130 | G | A | G |
| *qDF_3.1_* | *LOC_Os03g0762000_OsHD6* | 3 | 31744512 | G | T | G |
| *qPH_3.1_* | *LOC_Os03g0837300_OsLTS1* | 3 | 35235775 | A | C | A |
|  |  | 3 | 35295694 | C | C | A |
| *qYLD_3.1_* | *LOC_Os03g0786400_OsDST* | 3 | 33713486 | A | C | A |
|  |  | 3 | 33970114 | C | T | C |
| *qYLD_7.1_* | *LOC_Os07g0681500_OsMED5_3* | 7 | 28942458 | C | T | C |
| *qYLD_12.1_* | *LOC_Os12g0477400_OsNAC139* | 12 | 17443323 | T | C | T |
| *qGW_5.1_* | *LOC_Os05g0154700_OsSRS3* | 5 | 3253516 | G | A | G |
| *qFe_9.1_* | *LOC_Os09g0511000_OsLysM-RLK10* | 9 | 19798292 | C | T | C |
| *qFe_12.1_* | *LOC_Os12g0476200_OsSWEET13* | 12 | 17443323 | T | C | T |
| *qZn_1.1_* | *LOC_Os01g0343300_OsGATA14* | 1 | 13711317 | C | T | C |
|  |  | 1 | 13777831 | T | C | T |
| *qZn_5.1_* | *LOC_Os05g0164800_OsZIP6* | 5 | 3827315 | T | C | T |
| *qZn_9.1_* | *LOC_Os09g0511500* | 9 | 19967561 | A | G | A |


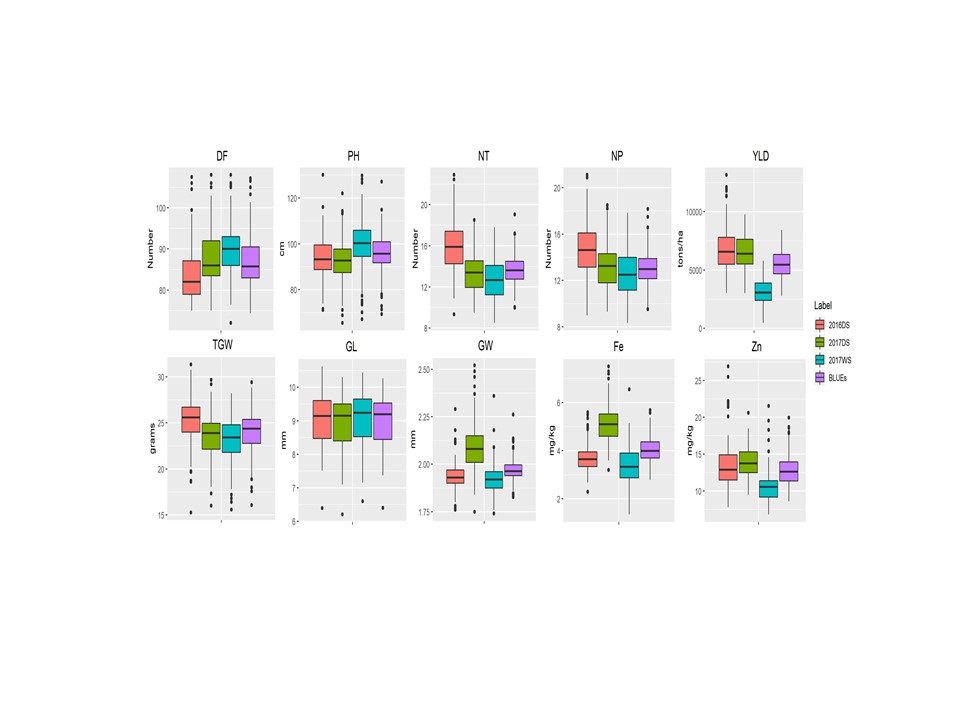


Figure S1. Box plots of agronomic traits and grain Fe and Zn concentrations using three seasons data and BLUEs in 148 DH lines of the cross IR05F102 × IR69428 evaluated under irrigated conditions during 2016DS, 2017DS, and 2017WS

† DF: days to flowering (days); PH: plant height (cm); NT: number of tillers; NP: number of panicles; YLD: yield (kgha-1); TGW: thousand grain weight (g); GL: grain length (mm); GW: grain width (mm); Fe: Iron (ppm); and Zn: Zinc (ppm). The box plot was generated using R core team^63^


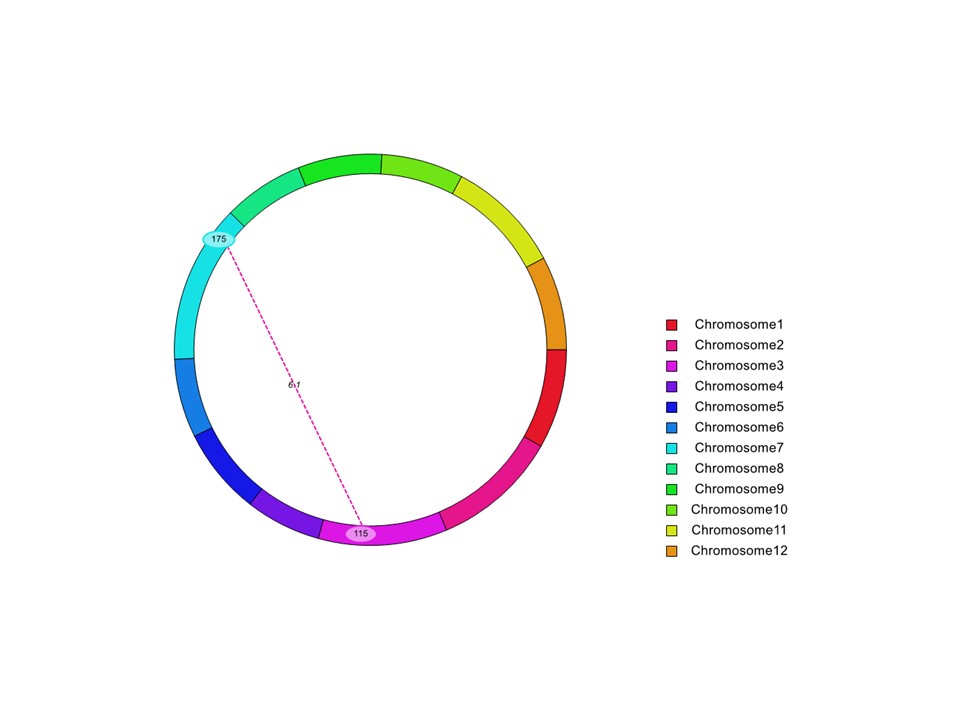


Figure S2. Cyclic illustration of epistatic QTLs for plant height identified in rice DH population.

The dotted lines indicate the interacting marker pairs located on the same or different chromosomes with corresponding LOD score due to epistatic effect.


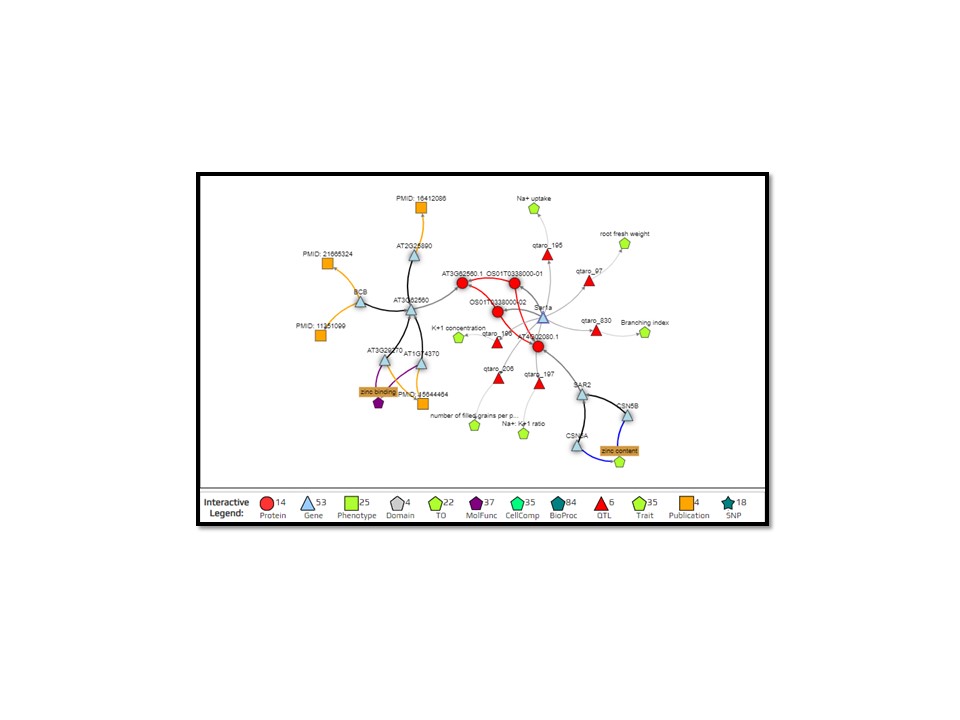


Figure S3 Candidate gene network showing the relationship between Sar1a


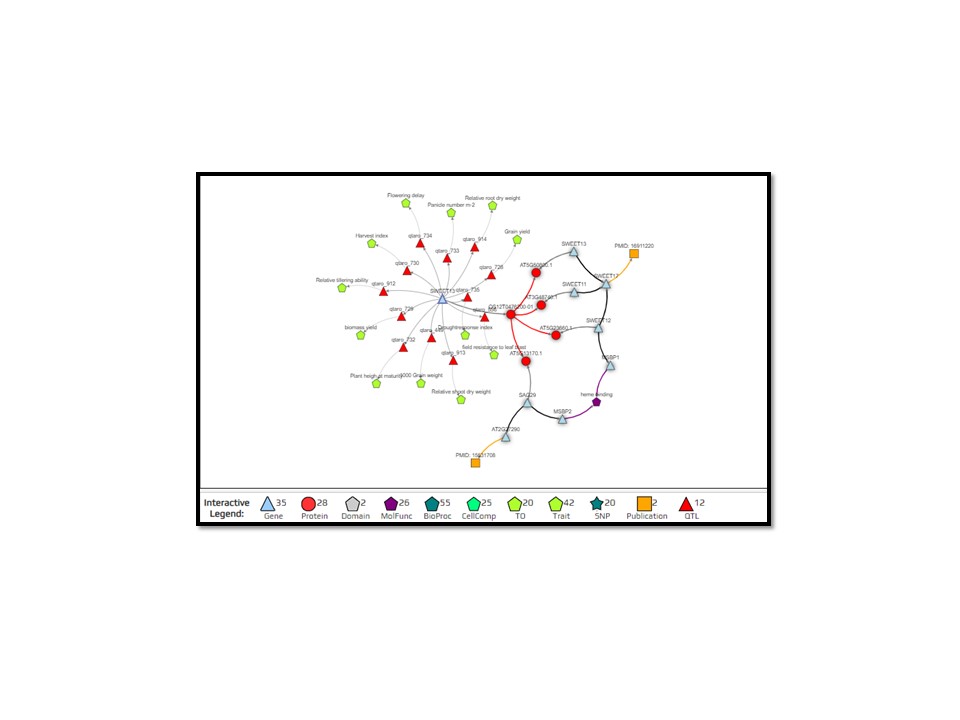


Figure S4. Candidate gene network showing the relationship between SWEET13

Note* Figures S3 and S4 were generated using the database K-netminer program^70^ (http://knetminer.rothamsted.ac.uk/ Oryza_sativa/).


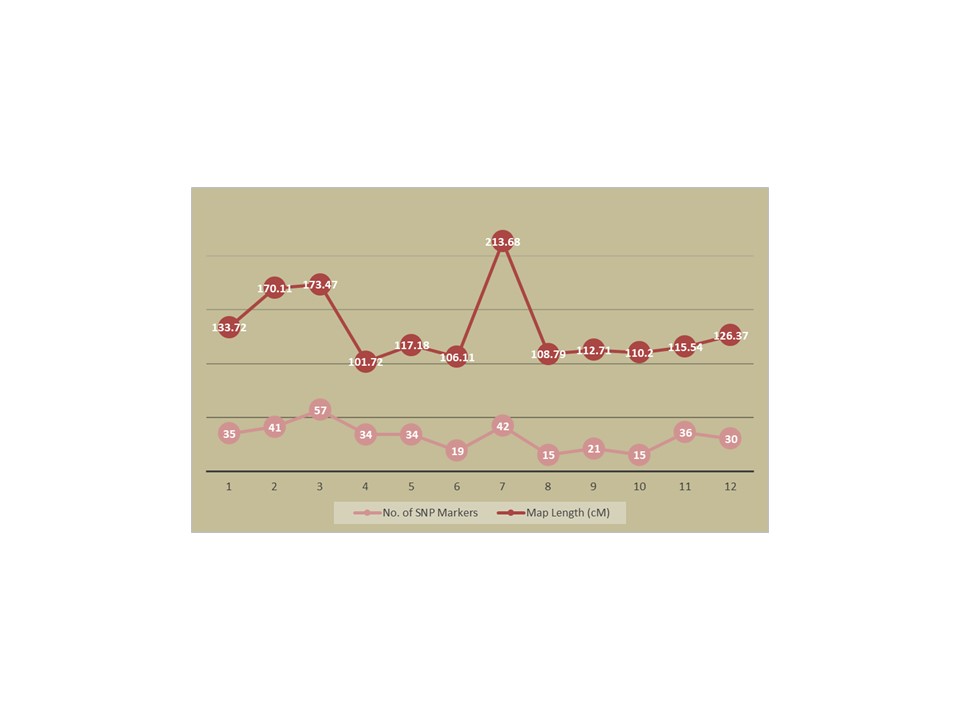


Figure S5. Chromosome wise SNP markers and genetic map length
